# Supplementary material for: Identification and Expression Profile of CLE41/44-PXY-WOX Genes in Adult Trees Pinus sylvestris L. Trunk Tissues during Cambial Activity
Source: Plants (Basel). 2023 Feb 13;12(4):835. doi: 10.3390/plants12040835 (PMC9961183; doi:10.3390/plants12040835)
Supplement: Supplementary file 1 [file plants-12-00835-s001.zip › Figure S1.pdf]

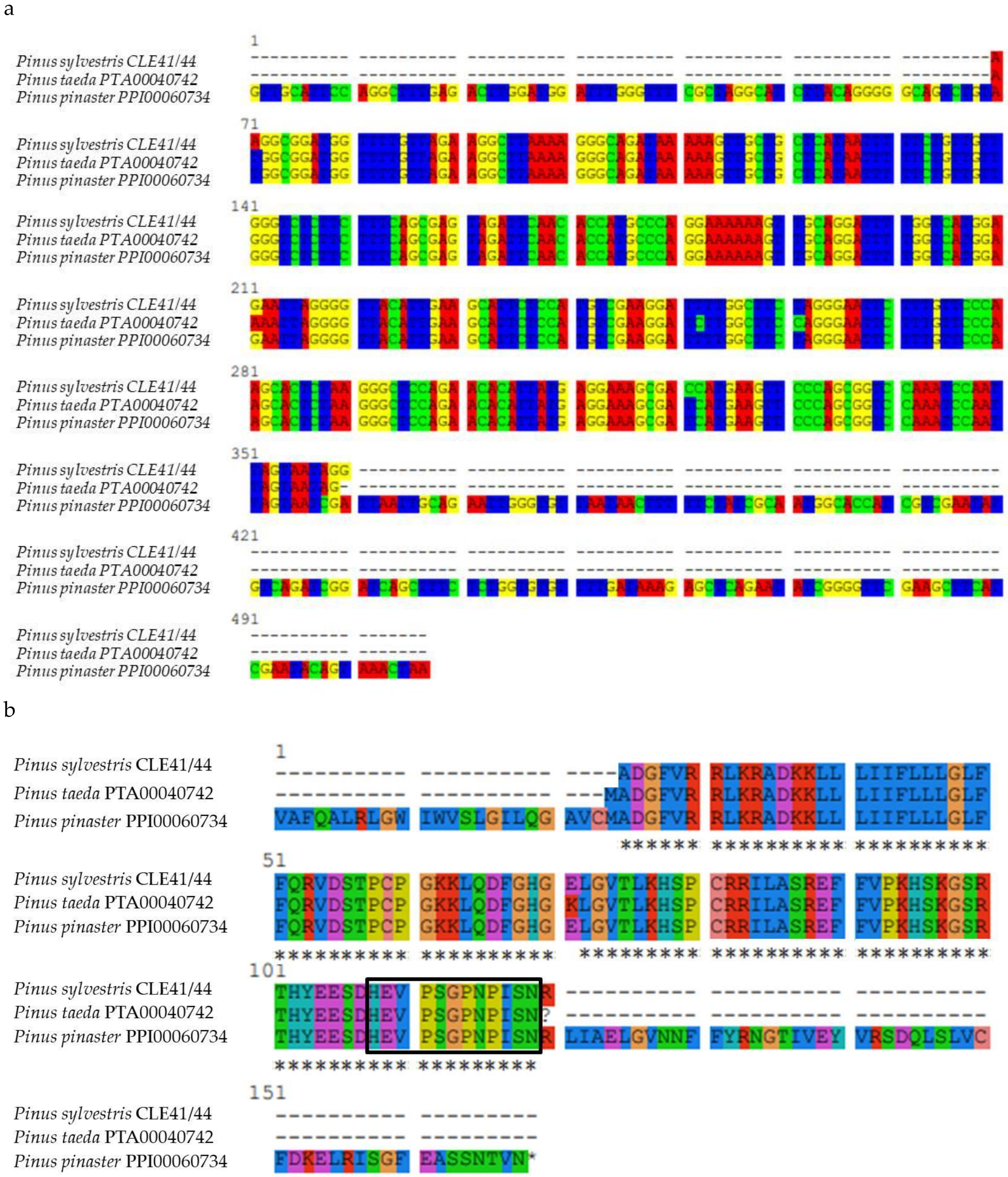

Supplementary Materials Figure S1: An alignment of the nucleotide (a) and amino acid (b) sequences of CLE41/44 from P. pinaster and P. taeda with the sequence derivated from P. sylvestris cloned DNA. Putative CLE peptide sequences are in black rectangle. Asterisks indicate identical amino acids. Alignment was performed using SeaView v.4 software.
