# Supplementary material for: Identification and Expression Profile of CLE41/44-PXY-WOX Genes in Adult Trees Pinus sylvestris L. Trunk Tissues during Cambial Activity
Source: Plants (Basel). 2023 Feb 13;12(4):835. doi: 10.3390/plants12040835 (PMC9961183; doi:10.3390/plants12040835)
Supplement: Supplementary file 1 [file plants-12-00835-s001.zip › Figure S2.pdf]

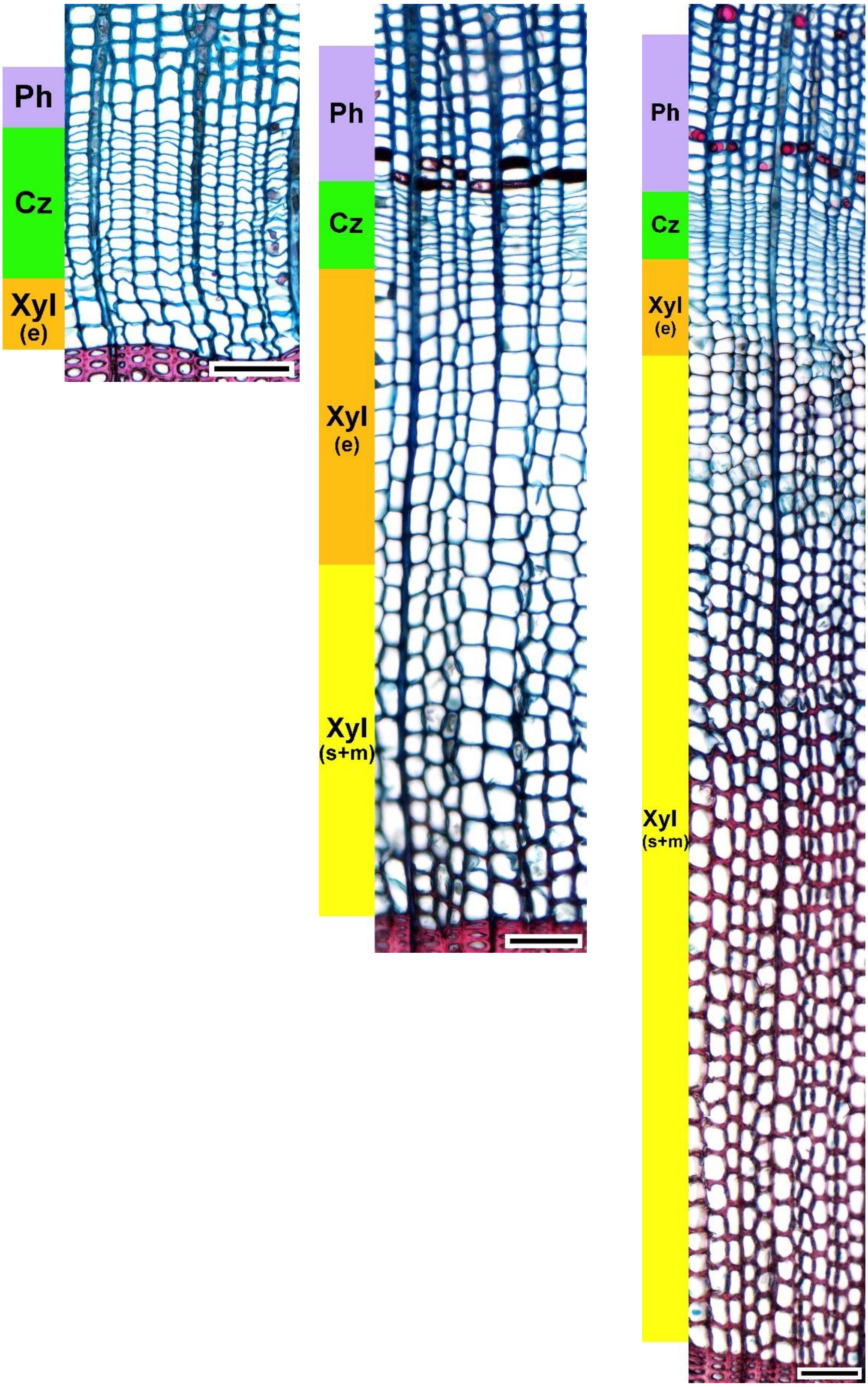

(a) (b) (c)

Supplementary Materials: Figure S2

Transverse trunk tissue sections include conductive phloem (Ph), cambial zone (Cz), expanding cells (Xyl (e)) and cells, which are forming secondary cell wall, and mature cells (Xyl (s+m)) of current year xylem in 40-year-old pine trees. Scale bar = 100 µm. Samples were collected on May 27 (a), June 21 (b) and July 21 (c), 2022.
