# Supplementary material for: Identification and Expression Profile of CLE41/44-PXY-WOX Genes in Adult Trees Pinus sylvestris L. Trunk Tissues during Cambial Activity
Source: Plants (Basel). 2023 Feb 13;12(4):835. doi: 10.3390/plants12040835 (PMC9961183; doi:10.3390/plants12040835)
Supplement: Supplementary file 1 [file plants-12-00835-s001.zip › Figure S3.pdf]

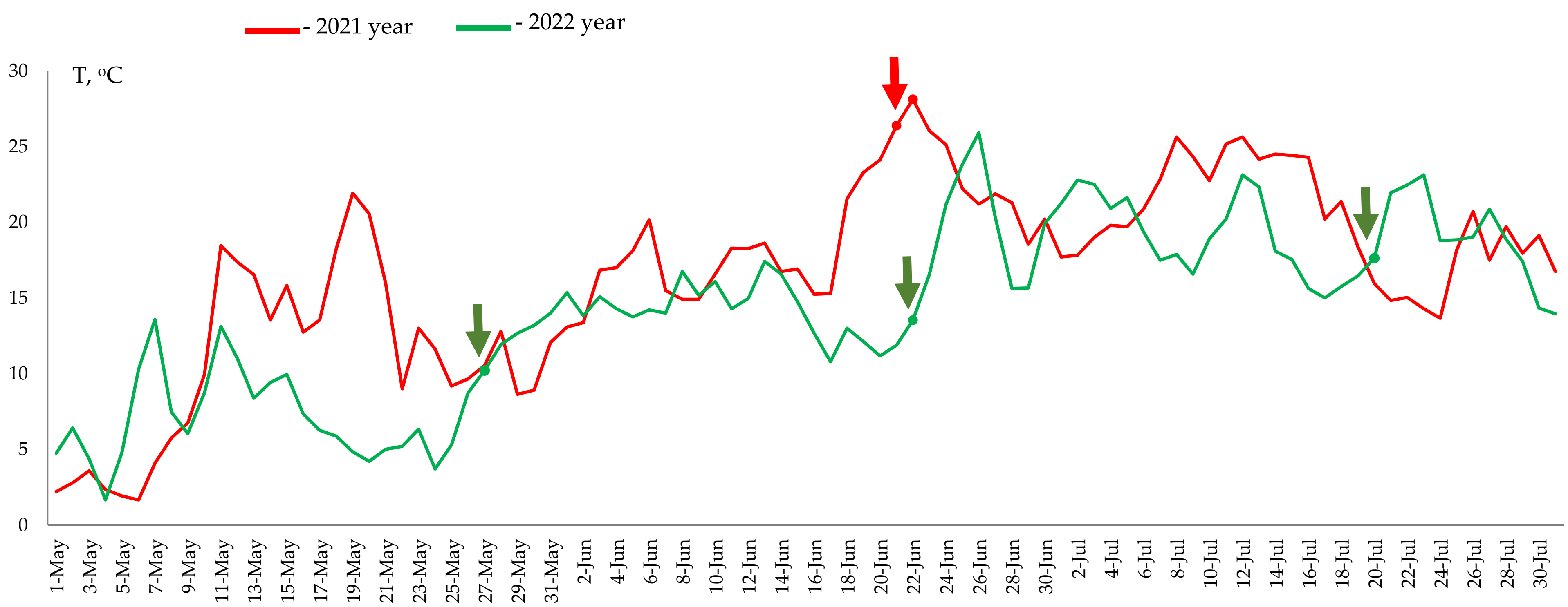

Supplementary Materials: Figure S3

The dynamics of daily average temperature during May-July 2021 and 2022 at the sampling sites. The arrows indicate sampling dates.
