# Supplementary material for: Identification and Expression Profile of CLE41/44-PXY-WOX Genes in Adult Trees Pinus sylvestris L. Trunk Tissues during Cambial Activity
Source: Plants (Basel). 2023 Feb 13;12(4):835. doi: 10.3390/plants12040835 (PMC9961183; doi:10.3390/plants12040835)
Supplement: Supplementary file 1 [file plants-12-00835-s001.zip › Figure S4.pdf]

(a)

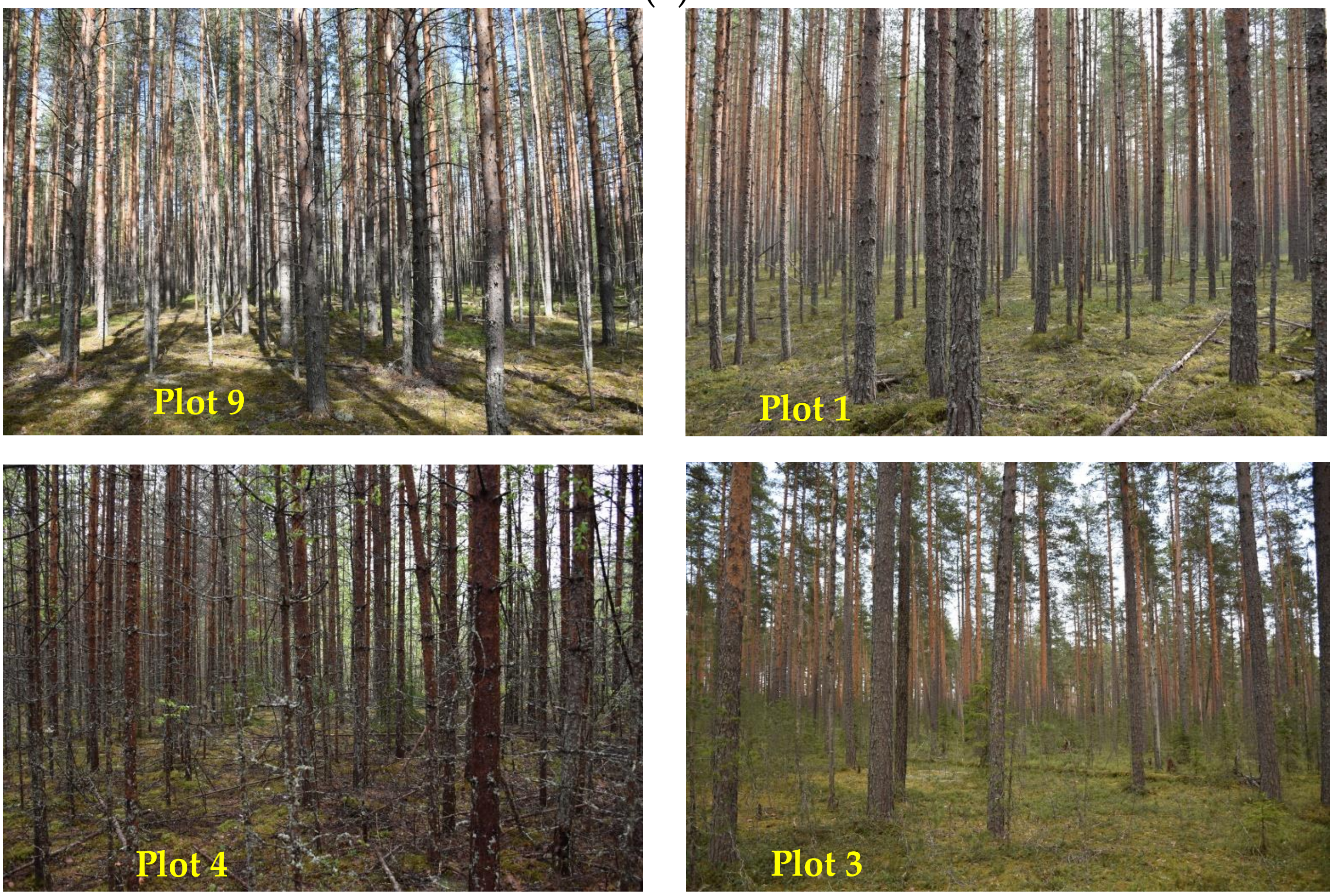

(b)

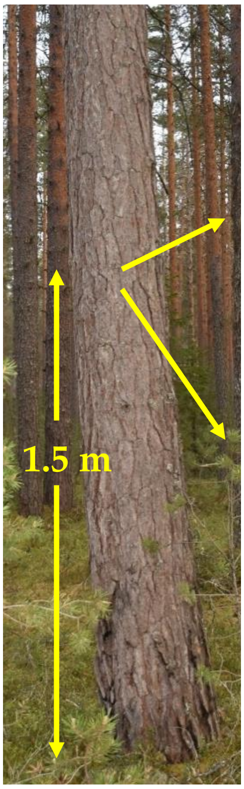

For molecular genetic analysis «windows» were cut out of the trunk and the bark was separated from the wood.

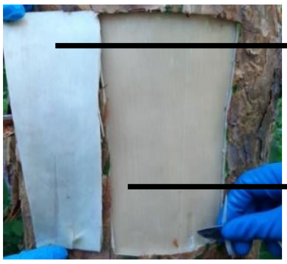

Fraction 1, prepared from the bark side, included cells of non-conductive phloem, fully formed early phloem, differentiating late phloem, and cambial zone.

Fraction 2, prepared from the debarked trunk surface, included differentiating early xylem cells.

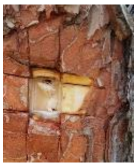

For microscopic analysis blocks (1 x 1 x 0.8 cm, length x width x height) were cut out of the trunk.

Supplementary Materials: Figure S4

The appearance of a 70- (Plot 9), 80- (Plot 1), 30- (Plot 4) and 180- (Plot 3) year-old lingonberry pine forest (a). Trunk tissue sampling plan for molecular genetics and microscopic analysis (b).
